# Impact of vitamin A on aged people’s cognition and Alzheimer’s disease progression in an animal model

**DOI:** 10.1038/s41538-025-00402-1

**Published:** 2025-05-08

**Authors:** Pengfei Li, Jingjing Xu, Yujie Guo, Xiaojun Ma, Xixiang Wang, Lu Liu, Yu Liu, Xiuwen Ren, Jiahao Li, Ying Wang, Liping Meng, Shaobo Zhou, Linhong Yuan

**Affiliations:** 1 School of Public Health, Capital Medical University, Beijing, 100069 China; 2 Beijing Key Laboratory of Environment and Aging, Capital Medical University, Beijing, 100069 China; 3 Suzhou Research Center of Medical School, Suzhou Hospital, Affiliated Hospital of Medical School, Nanjing University, Suzhou, 215153 Jiangsu China; 4 Inner Mongolia Mengniu Dairy (Group) Co., LTD., Hohhot, 011500 Inner Mongolia China; 5 School of Science, Faculty of Engineering and Science, University of Greenwich, Central Avenue, Chatham, ME4 4TB UK

**Keywords:** Malnutrition, Neuroimmunology

## Abstract

The relation between vitamin A (VA) level and cognitive function and the underlying mechanisms have not been thoroughly investigated. Population-based cross-sectional and animal diet intervention studies were conducted to analyze the association between VA nutritional status and cognitive function and the underlying mechanisms. In the population-based study, information from 1817 adults aged 50 years and above was used for data analysis, and we found that subjects with plasma VA level greater than 0.539 μg/ml displayed a lower risk of mild cognitive impairment (MCI). In the animal experiment, VA metabolism was disrupted in Alzheimer’s disease (AD) model mice, indicated by increased hepatic VA level and reduced retinol binding protein 4 (RBP4) level. AD model mice fed with low-VA diet showed worse nesting behavior, and cerebral pathologies, including increased Aβ generation, exacerbated neuroinflammation, and impaired brain glucose uptake and insulin signaling pathway. In conclusion, higher plasma VA level (≥ 0.539 μg/ml) might decrease the risk of MCI in the middle-aged and elderly individuals. Low VA nutritional status might disrupt brain glucose metabolism through regulating the insulin signaling pathway, promoting the senile plaque deposit and aggregating cerebral neuroinflammation, finally exacerbating the pathology of AD.

## Introduction

Cognitive impairment affects the well-being and quality of life of elderly individuals. It has emerged as a primary factor contributing to dependency, disability, and mortality in the aging population^1,2^. The global acceleration of population aged 60 years and older will reach 2.1 billion by 2050^3^. In China alone, by 2030, 25% of the population will be aged individuals^4^. As a result, cognitive decline and the increased risk of suffering from dementia become an urgent public health concern in China. Alzheimer’s disease (AD) is the most common cause of dementia, accounting for approximately 50–75% of dementia cases, as a progressive neurodegenerative disorder characterized by cognitive decline, memory loss, and impaired executive function^1^. Therefore, preventing the onset of cognitive decline and AD dementia is becoming a formidable challenge in China.

Vitamin A (VA), which includes retinol, retinoic acid, and retinaldehyde, represents a class of fat-soluble bioactive substances^5^. VA is essential for maintaining normal vision, tissue cell growth and differentiation, immune system function, and antioxidant capacity, and it plays a role in the formation of the central nervous system, and bone and embryo development. Besides, retinol was also reported to participate in the pathophysiological processes of cardiovascular disease, diabetes, cancer and obesity^6^. In addition, VA may be also an essential nutrient for maintaining the normal biological function of the central nervous system. Population-based studies have shown a positive correlation between dietary β-carotene intake, serum β-carotene level, and learning, memory and cognitive function in the elderly^7^. Compared to those with normal cognition, patients with mild to moderate cognitive impairment exhibited significantly reduced circulating VA level^8^. Additionally, decreased retinoic acid receptor expression was observed in the neocortex of AD patients^9^. Furthermore, a negative correlation between dietary VA intake and cerebral beta-amyloid (Aβ) deposition was found in a study of individuals aged 70 years and above^10^. All these data suggested that reasonable VA nutritional status might play a protective role against the development of AD.

Indeed, patients with AD showed defects in both transport and function of VA in the brain, as well as lower circulating or cerebrospinal VA level^11,12^, demonstrating that VA malnutrition or deficiency may lead to cognitive decline and increase the risk of AD in older individuals. VA dietary intervention delayed the progression of dementia in AD model mice^8^ by enhancing synaptic plasticity, and improving hippocampal neurogenesis and memory deficits^13,14^. These results indicate the potential role of VA in antagonizing the pathology and development of cognitive impairment and dementia. However, the precise relationship between VA nutritional status and cognitive function in middle-aged and elderly individuals remains unclear. Additionally, the effect and mechanism of low VA nutritional status on the occurrence and development of AD are not well understood.

The exact etiology of AD remains incompletely understood, but growing evidence suggested that metabolic dysfunctions, particularly related to glucose metabolism, play a crucial role in AD pathogenesis^15,16^. Recent studies have also highlighted the potential impacts of micronutrient deficiencies, such as VA, on cognitive function and metabolic disorder^17^. Data from our previous works also demonstrated a complex relation between blood VA level and the risk of diabetes^18^. The neuroprotective impact of VA and its metabolites has been implicated by previous studies, suggesting that VA deficiency may exacerbate cognitive decline, potentially influencing the onset and progression of neurodegenerative diseases^19^. Blood glucose metabolism, which refers to the body’s ability to utilize glucose effectively, is another critical factor for determining normal brain function^20^. Disturbed glucose metabolism in the brain has been linked to cognitive deficits and may contribute to the pathophysiology of AD^15,20^. Additionally, insulin resistance and dysregulated glucose metabolism have been shown to influence cerebral Aβ deposition and tau phosphorylation^21^. Given the potential interaction of VA, glucose metabolism, and neurodegeneration, we therefore speculated that VA deficiency could exacerbate glucose dysregulation, thereby accelerating cognitive decline and the progression of AD.

Therefore, in the present study, a community population-based cross-sectional study was conducted to explore the association between plasma VA levels and cognitive performance in the middle-aged and elderly individuals. Moreover, in order to uncover the mechanism of VA nutritional status on AD pathology, a dietary VA interventional study was conducted in APP/PS1 model mice and C57BL/6J wild-type mice. This study will provide essential reference data for bridging the gap between VA nutritional status and the dietary prevention of AD in the elderly.

## Results

### Demographics of the participants

As shown in Table 1, the average age of the participants was 66.3 ± 6.2 years. The average age of subjects in the mild cognitive impairment (MCI) group was higher than that of the control group (*P* < 0.001). The educational level of MCI group was significantly lower than that of control group (*P* < 0.001). The proportion of individuals with daily physical activity habit in the control group was higher than that in the MCI group (*P* < 0.05). The percentage of subjects with reading habits was significantly higher in the control group than that in the MCI group (*P* < 0.001), and the percentage of individuals taking dietary supplements in the control group was lower than that in the MCI group (*P* < 0.05). The proportion of subjects with stroke and chronic kidney disease history in MCI group was significantly higher than that in the control group (*P* < 0.01). The MCI group showed higher fasting blood glucose (FBG) and plasma high-density lipoprotein cholesterol (HDL-c) levels than the control group (*P* < 0.05).

**Table 1:** Demographic and dietary intakes of the participants

Variables/ Characteristic	MCI (*n* = *679*)	Control (*n* = *1138*)	Total (*n* = *1817*)	*P* value
Age (year)	67.2 ± 6.8	65.8 ± 5.8	66.3 ± 6.2	< 0.001
Gender, n (%)				
*Male*	240 (35.3)	372 (32.7)	612 (33.7)	0.246
*Female*	439 (64.7)	766 (67.3)	1205 (66.3)	
BMI (kg/m^2^)	25.0 ± 3.3	25.1 ± 3.4	25.0 ± 3.4	0.548
Education, n (%)				
*Illiterate*	30 (4.4)	67 (5.9)	97 (5.3)	< 0.001
*Primary school*	106 (15.6)	210 (18.5)	316 (17.4)	
*Junior high school*	327 (48.2)	420 (36.9)	747 (41.1)	
*High school*	163 (24.0)	300 (26.4)	463 (25.5)	
*Junior college*	33 (4.9)	95 (8.3)	128 (7.0)	
*Undergraduate and above*	20 (2.9)	46 (4.0)	66 (3.6)	
Physical activity, n (%)				
*Never*	53 (7.8)	77 (6.8)	130 (7.2)	0.037
*1–3 days/week*	56 (8.2)	129 (11.3)	185 (10.2)	
*4–6 days/week*	57 (8.4)	124 (10.9)	181 (10.0)	
*Everyday*	513 (75.6)	808 (71.0)	1321 (72.7)	
Smoking, n (%)				
*Never*	510 (75.1)	843 (74.1)	1353 (74.5)	0.598
*Ever*	80 (11.8)	127 (11.2)	207 (11.4)	
*Current*	89 (13.1)	168 (14.8)	257 (14.1)	
Alcohol drinking (yes), n (%)	175 (25.8)	305 (26.8)	480 (26.4)	0.631
Living alone (yes), n (%)	51 (7.5)	70 (6.2)	121 (6.7)	0.261
Reading (yes), n (%)	238 (35.1)	503 (44.2)	741 (40.8)	< 0.001
TV and computer using (yes), n (%)	649 (95.6)	1097 (96.4)	1746 (96.1)	0.385
Dietary supplement (yes), n (%)	183 (27.0)	250 (22.0)	433 (23.8)	0.015
AD family history (yes), n (%)	56 (8.2)	103 (9.1)	159 (8.8)	0.558
Hyperlipidemia (yes), n (%)	292 (43.0)	492 (43.2)	784 (43.1)	0.924
Stroke (yes), n (%)	72 (10.6)	65 (5.7)	137 (7.5)	< 0.001
Chronic kidney disease (yes), n (%)	55 (8.1)	54 (4.7)	109 (6.0)	0.004
Plasma VA, (μg/ml)	0.49 ± 0.14	0.55 ± 0.17	0.53 ± 0.17	< 0.001
Nutritional VA status, n (%)				
*Deficiency*	3 (0.4)	3 (0.3)	6 (0.3)	0.372
*Marginal deficiency*	34 (5.0)	43 (3.8)	77 (4.2)	
*Sufficiency*	642 (94.6)	1092 (96.0)	1734 (95.4)	
Plasma parameters, (mmol/L)				
*FBG*	5.92 ± 1.78	5.72 ± 1.74	5.79 ± 1.76	0.018
*TC*	5.03 ± 1.04	4.96 ± 1.01	4.99 ± 1.02	0.168
*TG*	1.70 ± 1.16	1.76 ± 1.31	1.73 ± 1.25	0.342
*HDL-c*	1.45 ± 0.30	1.42 ± 0.32	1.43 ± 0.31	0.044
*LDL-c*	2.90 ± 0.86	2.96 ± 0.89	2.93 ± 0.88	0.175
Daily dietary intake, (g/d)				
*Cereal*	275.0 (175.0, 325.0)	250.0 (175.0, 300.0)	250.0 (175.0, 325.0)	0.545
*Fruit*	125.0 (75.0, 225.0)	125.0 (75.0, 225.0)	125.0 (75.0, 225.0)	0.471
*Vegetable*	275.0 (175.0, 425.0)	325.0 (175.0, 425.0)	275.0 (175.0, 425.0)	0.043
*Legume*	26.8 (16.1, 44.6)	26.8 (16.1, 44.6)	26.8 (16.1, 44.6)	0.083
*Whole grain*	26.8 (16.1, 62.5)	26.8 (16.1, 44.6)	26.8 (16.1, 53.6)	< 0.001
*Red meat*	26.8 (16.1, 44.6)	26.8 (16.1, 44.6)	26.8 (16.1, 44.6)	0.120
*Poultry meat*	16.1 (0, 26.8)	16.1 (0, 16.1)	16.1 (0, 17.9)	0.944
*Dairy*	128.6 (50.9, 214.3)	114.3 (42.9, 188.6)	125.0 (42.9, 214.3)	0.017
*Fish*	16.1 (0, 26.8)	16.1 (5.4, 26.8)	16.1 (5.4, 26.8)	0.888
*Egg*	42.9 (21.4, 53.6)	32.1 (21.4, 50.0)	35.7 (21.4, 50.0)	< 0.001
*Cooking oil*	25.6 (17.0, 38.3)	25.6 (17.0, 38.3)	25.6 (17.0, 38.3)	0.939
Dietary balance index				
*LBS*	15.0 (11.0, 21.0)	16.0 (12.0, 21.0)	16.0 (11.0, 21.0)	0.042
*HBS*	5.0 (2.0, 10.0)	5.0 (2.0, 8.0)	5.0 (2.0, 8.5)	< 0.001
*DQD*	22.0 (17.0, 27.0)	22.0 (17.0, 27.0)	22.0 (17.0, 27.0)	0.515

Continuous data were expressed as mean ± standard deviation (SD) or median (quartile 25th, quartile 75th), and student’s *t*-test and Mann-Whitney U test were used for comparison between groups. Categorical data were expressed as number and percentage, and chi-square test and Fisher’s exact test were used for comparison between groups.

*FBG* fasting blood glucose, *LBS* low bound score, *HBS* high bound score, *DQD* diet quality distance.

The subjects in MCI group consumed more whole grain, dairy, and egg, but fewer vegetables than the subjects in control group (*P* < 0.05). The MCI group has significantly lower low bound score (LBS), but higher high bound score (HBS) than the control group (*P* < 0.05) (Table 1).

### Plasma VA level in MCI and control subjects

**Fig. 1:**
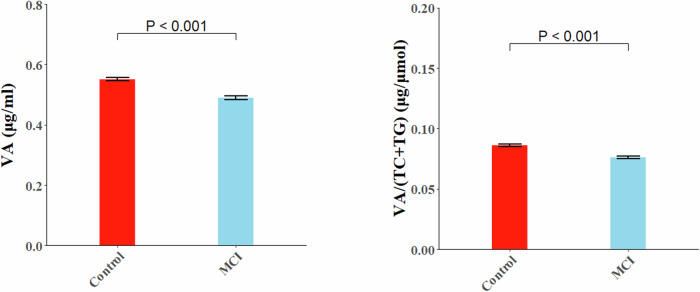
Comparison of plasma VA and lipid-adjusted VA levels between MCI and control groups. General linear model was used to compare plasma VA and lipid-adjusted VA levels between groups, adjusting age, gender, BMI, physical activity, smoking, drinking alcohol, usage of dietary supplement, hyperlipidemia (not adjusted if compared plasma VA/(TC + TG) levels), stroke, chronic kidney disease, T2DM, LBS, and HBS.

The average plasma VA concentration of the total participants was 0.53 ± 0.17 μg/ml, but it was significantly lower in the MCI subjects (0.49 ± 0.14 μg/ml) than the control ones without cognitive issue (0.55 ± 0.17 μg/ml) (*P* < 0.001) (Table 1). After further adjusting covariates in General Linear Model (GLM) analysis, both plasma VA and lipid-adjusted VA levels calculated by VA/[total cholesterol(TC)+triglyceride(TG)] were decreased significantly in MCI subjects as comparing with the control subjects (*P* < 0.001) (Fig. 1).

### Prevalence of MCI according to the plasma VA quintiles

The range of plasma VA among all subjects was divided into 5 quintiles, from quintile 1 (Q1), lowest 20% to Q5, the highest 80-100%. The prevalence of MCI according to the quintiles of plasma VA and lipid-adjusted VA levels was analyzed. Results are shown in Table 2. There were significant differences in the prevalence of MCI across the quintiles of plasma VA and lipid-adjusted VA levels (*P* < 0.001). The prevalence of MCI displayed a gradually decreasing trend from Q1 to Q5 quintile, and the subjects with Q5 level of both plasma VA and lipid-adjusted VA had the lowest prevalence of MCI than other groups (*P* < 0.001).

**Table 2:** Prevalence of MCI according to the quintiles of plasma VA and lipid-adjusted VA levels

Plasma Indexes	MCI	Q1	Q2	Q3	Q4	Q5	*χ*^2^	*P* value
VA	Yes	174 (47.7)	146 (40.2)	164 (45.1)	126 (34.7)	69 (19.1)	79.941	<0.001
	No	191 (52.3)	217 (59.8)	200 (54.9)	237 (65.3)	293 (80.9)		
VA/(TC + TG)	Yes	173 (46.1)	159 (44.9)	140 (37.5)	120 (34.0)	87 (24.0)	50.150	<0.001
	No	202 (53.9)	195 (55.1)	233 (62.5)	233 (66.0)	275 (76.0)		

Data were showed as number and percentage. The chi-square test was performed to compare the difference of MCI prevalence across quintiles.

*Q* quintile, *TC* total cholesterol, *TG* triglyceride.

### Cognition according to plasma VA level

**Fig. 2:**
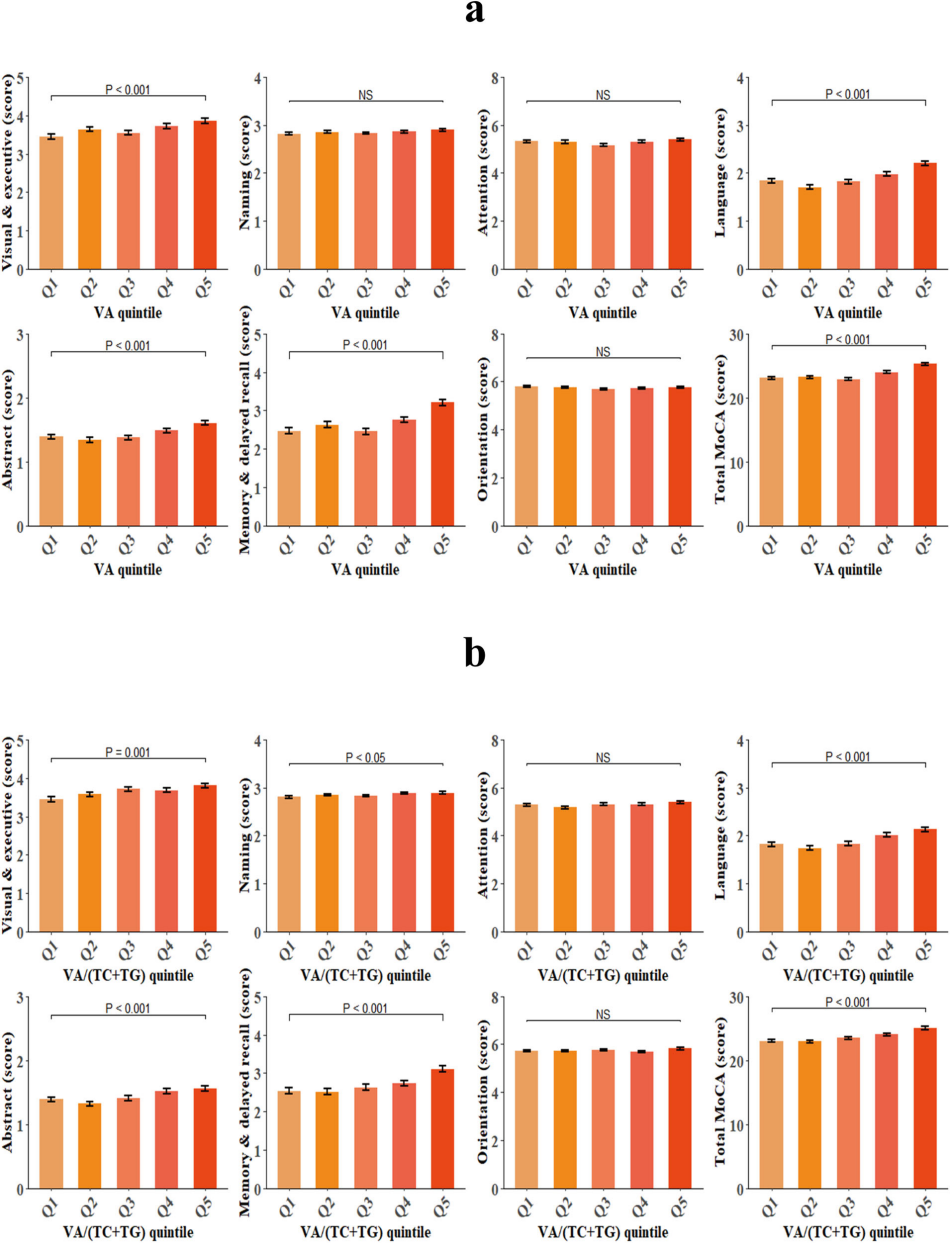
Cognitive functions between plasma VA levels. **a** Cognitive function across the quintiles of plasma VA levels. **b** Cognitive function across the quintiles of plasma VA/(TC + TG) levels. General linear model was applied to compare the cognitive functions between groups, adjusting age, gender, BMI, education, physical activity, living alone, reading habits, using TV and computer, smoking, drinking alcohol, usage of dietary supplements, AD family history, hyperlipidemia (not adjusted if compared between quintiles of plasma VA/(TC + TG) levels), stroke, chronic kidney disease, T2DM, LBS, and HBS.

Cognitive performance in all domains of Montreal Cognitive Assessment (MoCA) was analyzed. The significant differences in cognition across the quintiles of plasma VA or lipid-adjusted VA level were observed, and the statistical significance was mainly observed in visual & executive, language, abstract, and memory & delayed recall domains, and total MoCA score (*P* ≤ 0.001). In addition, subjects with the highest quintile of plasma VA or lipid-adjusted VA level displayed the best cognitive performance in these domains (Fig. 2a, b).

### The association of plasma VA level with the risk of MCI

**Fig. 3:**
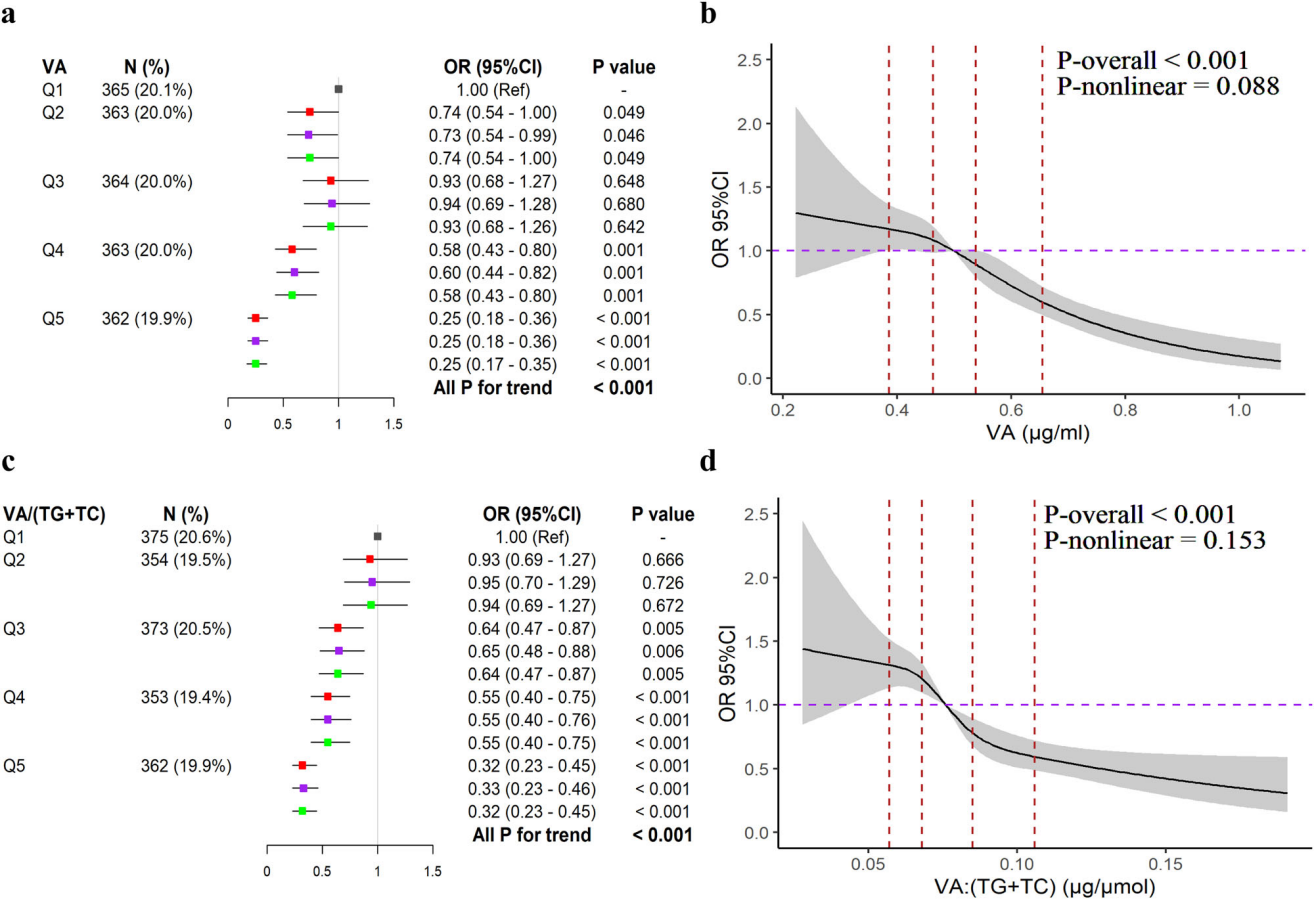
The association between plasma VA levels and MCI. **a** Forest plot for relation between plasma VA level and the risk of MCI. **b** Dose-response relation between plasma VA level and the risk of MCI. **c** Forest plot for relation between plasma VA:(TC + TG) level and the risk of MCI. **d** Dose-response relation between plasma VA:(TC + TG) level and the risk of MCI. Logistic regression analysis was used to analyze the association between plasma VA or VA: (TC + TG) level and risk of MCI. Logistic regression was adjusted by age, gender, BMI, education, physical activity, living alone, reading habits, using TV and computer, smoking, drinking alcohol, usage of dietary supplements, AD family history, hyperlipidemia (not adjusted for the plasma VA/(TC + TG) level), stroke, chronic kidney disease, and T2DM in model 1. Model 2 was further adjusted by LBS and HBS in model 1. Model 3 was further adjusted by DQD in model 1. Restricted cubic spline analysis was used to analyze the dose-response relationships with four knots at the 20th, 40th, 60th, and 80th percentiles of plasma VA or VA/(TC + TG) levels, adjusted by covariates in model 2. In Fig. 4a, c, the red, purple, and green dots were represented of model 1, model 2, and model 3, respectively. In Fig. 4b, d, the four vertical lines indicated the 20th, 40th, 60th, and 80th percentiles of plasma VA or VA/(TC + TG) levels, respectively.

To accurately analyze the plasma VA level in relation to the risk of MCI, two statistical models were applied. As shown in Fig. 3a, the plasma VA in subjects from the Q2 (VA: 0.387 - 0.463 μg/ml), Q4 (VA: 0.539–0.655 μg/ml), and Q5 (VA ≥ 0.656 μg/ml) groups have a lower risk of MCI than those from the Q1 (VA ≤ 0.386 μg/ml) group (*P* < 0.05). There was a linear decreasing trend in the risk of MCI from Q1 to Q5 group (*P*_*trend*_ < 0.001) (top of the left panel). The linear association between plasma VA level and the risk of MCI was also demonstrated by the restricted cubic spline (RCS) curve (top of the right panel), especially when the plasma VA level was ≥ 0.539 μg/ml (*P*_*overall*_ < 0.001; *P*_*nonlinear*_ > 0.05) (Fig. 3b; Supplemental Table S1). Thus, the marginal plasma VA level, in this study, over 0.539 μg/ml, was recommended to reduce the MCI risk.

A similar negative association of plasma VA/(TC + TG) with the risk of MCI was also found in this study. Subjects with Q3 (0.069–0.085 μg/μmol) to Q5 ( ≥ 0.107 μg/μmol) level of lipids-adjusted VA showed a significantly decreased risk for MCI (*P* < 0.01). The results of the trend test further demonstrated that the increase in VA/(TC + TG) level was a protective factor for MCI (*P*_*trend*_ < 0.001) (Fig. 3c, bottom). RCS analysis also showed a linear decrease in the risk of MCI according to the increase of lipids-adjusted VA level (*P*_*overall*_ < 0.001, *P*_*nonlinear*_ > 0.05), especially from Q2 (0.058 - 0.068 μg/μmol) to Q5 ( ≥ 0.107 μg/μmol) group (Fig. 3d; Supplemental Table S1).

### Impact of VA nutritional status on dietary intake and body weight of mice

**Fig. 4:**
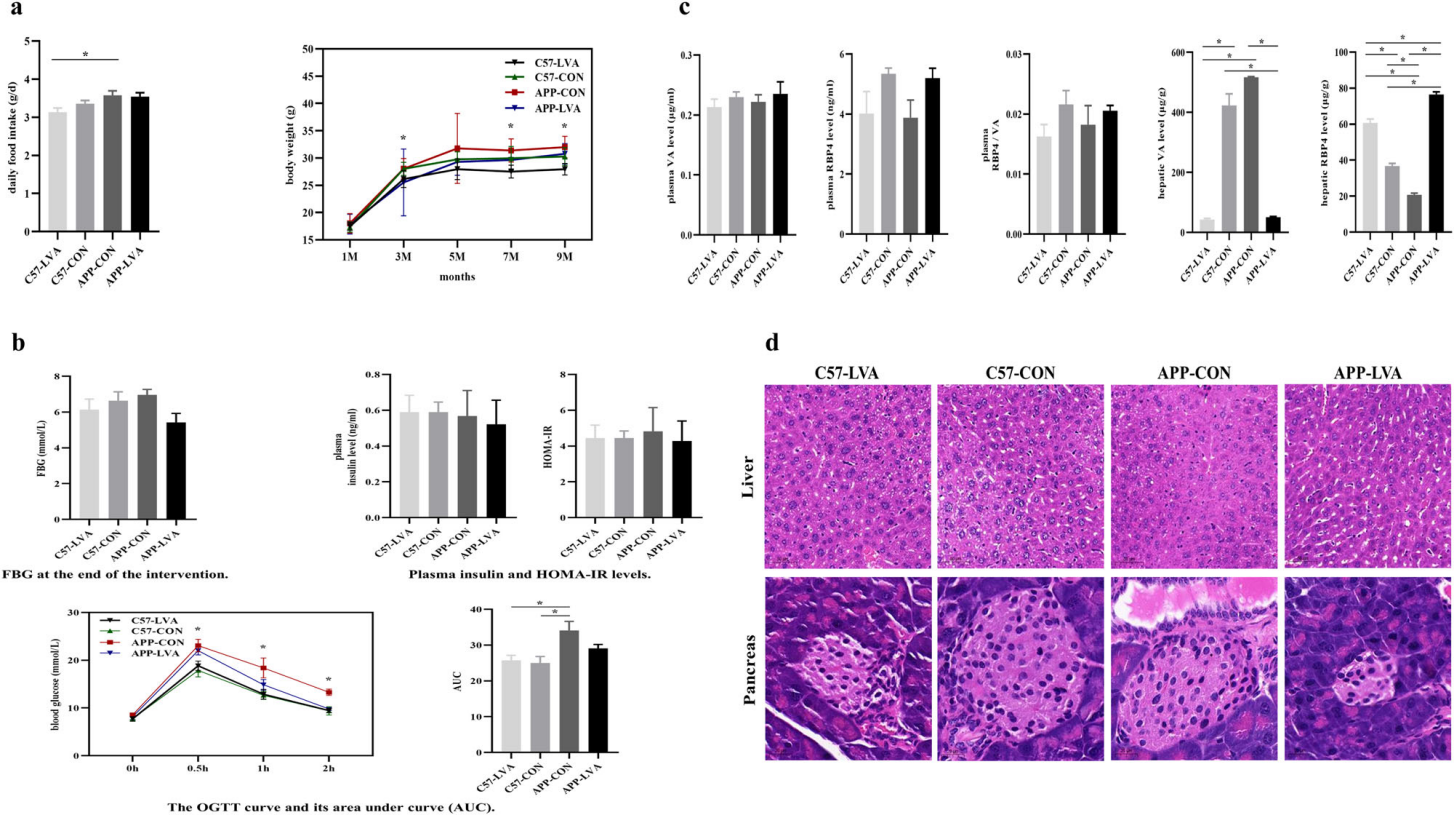
Growth of animals, glucose and VA metabolism, and hepatic and pancreatic histology. **a** Dietary intake and body weight monitoring of C57BL/6J and APP/PS1 mice during the dietary intervention. **b** FBG and plasma insulin levels, HOMA-IR, and OGTT at the end of the intervention. **c** Plasma VA and RBP4 levels and the ratio of RBP4 to VA, hepatic VA and RBP4 levels at the end of the intervention. **d** HE staining of the liver (scale bar: 50μm) and the pancreas (scale bar: 20μm) in mice treated with different diets at the end of the intervention. Data were expressed as mean ± SEM or mean ± SD, n = 8 in C57-CON group, n = 8 in C57-LVA group, n = 8 in APP-CON group, n = 6 in APP-LVA group. Comparisons between groups were conducted using one-way ANOVA, followed by $\check{{\rm{S}}}{\rm{id}}\acute{{\rm{a}}}{\rm{k}}$ or Games-Howell multiple comparisons test according to the homogeneity of variance. *P < 0.05. Asterisks among all histogram figures indicated significant difference of post-hoc comparison between two groups at two ends of the horizontal line, except the comparison of body weight in Fig. 5a and the comparison of OGTT test at different time points in Fig. 5b (Here asterisks indicated the significant difference among four groups).

As shown in Fig. 4a, daily diet intake in both groups of APP/PS1 mice had slightly higher than the two groups of C57 mice. Food intake in mice fed low VA (LVA) diet was reduced as comparing with the animals fed with control diet among both C57 and APP/PS1 groups, but no significant difference (Fig. 4a, left). In addition, the mice from the APP-CON group showed significantly higher daily diet intake than C57-LVA mice (*P* < 0.05). There was a significant difference in body weight between the four groups at 7 and 9 months, respectively. The highest body weight was found in the APP-CON group, and the lowest body weight was observed in the C57-LVA group (*P* < 0.05) (Fig. 4a, right), as they consumed the highest and lowest amount of diet during the experimental period, respectively.

### Impact of VA nutritional status on glucose metabolism

As shown in Fig. 4b, there was not significant difference in FBG, plasma insulin and homeostasis model assessment of insulin resistance (HOMA-IR) levels between C57 and APP/PS1 control mice. Low VA dietary intervention did not obviously affect these parameters in C57 mice, but caused a decreased trend in APP/PS1 mice, although the difference was not statistically significant. In the oral glucose tolerance test (OGTT) (Fig. 4b, bottom, left), the blood glucose level was significantly different among four groups at 0.5 h and 1 h after glucose administration (*P* < 0.05), among which the blood glucose level of mice from APP-CON and APP-LVA groups was higher than that of C57 mice, especially, the mice from APP-CON group showed much higher glucose level. In addition, the mice from the APP-CON group had the highest area under the curve (AUC) than mice from other groups (*P* < 0.05) (Fig. 4b, lower right).

### Impact of VA nutritional status on plasma VA, hepatic VA, and retinol-binding protein 4 (RBP4) levels

Retinol binding protein 4 (RBP4), which is primarily synthesized and released from the adipose and liver, is the particular transporter protein for VA, regulating the circulating level of retinol^22^. As shown in Fig. 4c, although there was no significant difference in plasma VA and RBP4 levels, and ratio of RBP4/VA among groups, the APP/PS1 control mice showed relatively lower plasma RBP4 level and RBP4/VA ratio than C57 control mice. The treatment of low VA diet caused opposite effects on plasma RBP4 level and the RBP4/VA ratio in C57 and APP/PS1 mice, as demonstrated by a decreased trend in C57 mice, but an increased trend in APP/PS1 mice.

APP/PS1 control mice showed higher hepatic VA level but significantly lower RBP4 level than C57 control mice (*P* < 0.05). The treatment of a low VA diet significantly decreased hepatic VA level in both C57 and APP/PS1 mice (*P* < 0.05), accompanying with dramatically increased hepatic RBP4 level (*P* < 0.05). The low VA diet-fed APP/PS1 mice showed much higher increase in hepatic RBP4 level than C57-LVA mice (*P* < 0.05).

### Impact of VA nutritional status on hepatic and pancreatic histology

As shown in Fig. 4d, there was no difference in both hepatic and pancreatic histology between C57 and APP/PS1 control mice, but there was histological damage observed in the liver and pancreas in both C57 and APP/PS1 mice fed with low VA diet. In the liver, enlarged hepatocyte spaces and swollen hepatocytes were observed. Low-VA diet caused obviously pancreatic damage and decreased islet area in both C57 and APP/PS1 mice. In the pancreas of mice from APP-LVA group, several histological damages were observed in the islets, which were characterized by unclear borders with surrounding tissues, disordered cell arrangement, cell atrophy, and absent nuclei. Prominent pancreatic fibrosis was also observed in APP/PS1 mice fed with low VA diet.

### Impact of VA nutritional status on behavior of mice

**Fig. 5:**
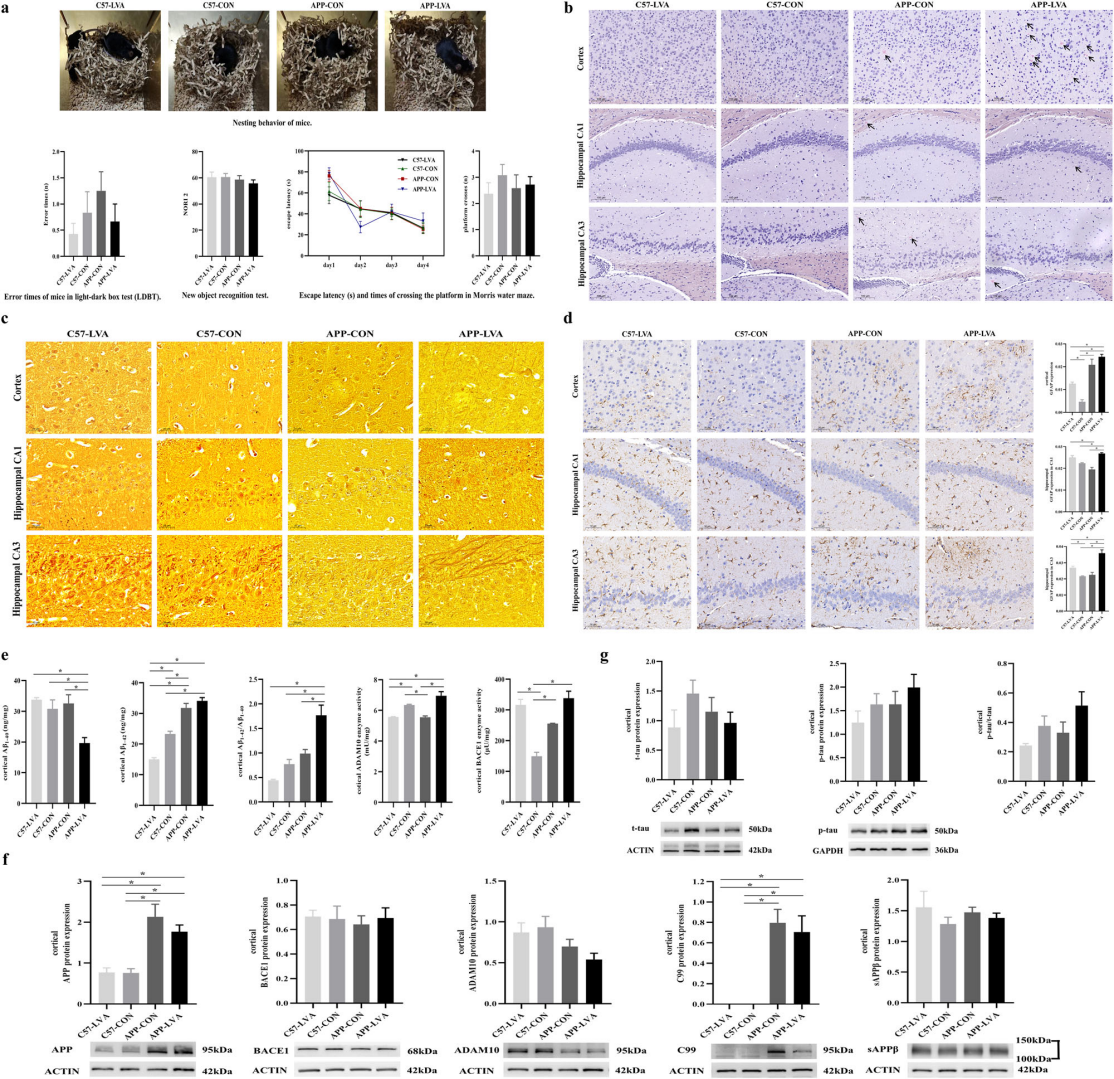
Behavioral tests, AD pathological changes, and cortical Aβ content and metabolism-related enzyme activity and molecule expression. **a** Results of behavior testing of mice at 9 months. **b** Results of senile plaque with Congo red staining of mice in the hippocampus and cortex. **c** Results of silver staining of mice in the hippocampus and cortex. **d** GFAP expression in the hippocampus and cortex among four groups. **e** Cortical Aβ content, ADAM10 and BACE1 enzyme activity. **f** Cortical APP, BACE1, ADAM10, C99, and sAPPβ expression. **g** Cortical t-tau and p-tau expression, and p-tau/t-tau ratio in mice treated with different diets. Data were expressed as mean ± SEM or mean ± SD, n = 8 in C57-CON group, n = 8 in C57-LVA group, n = 8 in APP-CON group, n = 6 in APP-LVA group. For Fig. 6b, the black arrow was used to indicate senile plaque. Comparisons between groups were conducted using one-way ANOVA, followed by $\check{{\rm{S}}}{\rm{id}}\acute{{\rm{a}}}{\rm{k}}$ or Games-Howell multiple comparisons test according to the homogeneity of variance. *P < 0.05. Asterisks among all histogram figures indicated significant difference of post-hoc comparison between two groups at two ends of the horizontal line.

The nesting behavior of animals was tested. As shown in Fig. 5a, the mice in APP-LVA group had poor nesting behavior, while, animals from other groups showed similar nesting behavior. In the light-dark box test (LDBT), the mice from the APP-CON group had the highest error time among four groups, and the low VA diet reduced the error time in both APP/PS1 and C57 mice, but the difference was not statistically significant. No difference between groups was observed in the new object recognition (NOR) and water maze tests.

### Impact of VA nutritional status on cortical and hippocampal amyloid plaque

Amyloid plaque was not detected in the brain of C57 mice, but observed in the cortex and hippocampal CA1 and CA3 regions in APP/PS1 mice fed with normal or low VA diet, especially a greater number of amyloid plaques were observed in the cortex of APP/PS1 mice fed with low VA diet (Fig. 5b).

The results of silver glycine staining were shown in Fig. 5c. Regular neuronal morphology and deeply stained nerve fibers were observed in control diet-fed C57 mice but not in the control diet-fed APP/PS1 mice, as irregular arrangements were found in the neuron of both the cortex and hippocampal CA1 region of APP-CON mice. Significant loss of neuron, atrophied neurons, nucleus deformation and senile plaques were also observed in the cortex and hippocampal CA1 region. Nucleolysis was also observed, and microglia infiltrated the area surrounding the injured neurons. Irregular cortical and hippocampal neuron arrangements were also observed in low VA diet-fed C57 mice, and this irregularity became much more serious in low VA diet-fed APP/PS1 mice. Loss of neuron in the cortex and hippocampal CA1 region was more significant in the low VA diet-fed APP/PS1 mice, and in these mice, the senile plaques were tightly surrounded by numerous brown nerve fibers, and a clear infiltration of microglial cells was observed.

### Impact of VA nutritional status on glial fibrillary acidic protein (GFAP) expression in the brain

In C57 control mice, branched astrocytes with normal size and uniform cell distribution were observed in the cortex and hippocampus. In Fig. 5d, immunohistochemistry (IHC) assay results showed that there were significantly greater cortical GFAP expression in APP/PS1 control mice than in C57 control mice (*P* < 0.05). Moreover, significant morphological changes, including typical activated astrocytes with shorter and thicker branches, were found in APP/PS1 control mice. Low VA diet caused a significant increase in cortical and hippocampal GFAP expression in both C57 and APP/PS1 mice, and the difference between groups was statistically significant (*P* < 0.05). Increased GFAP expression was mainly observed in the cortex and hippocampal CA3 domain in low VA diet-fed C57 mice (*P* < 0.05). In APP/PS1 mice, deformed astrocytes heavily aggregated around the amyloid plaque in both the cortex and hippocampus, and the significantly increased GFAP expression was found in the hippocampal CA1 and CA3 domains of low VA diet-fed APP/PS1 mice (*P* < 0.05) (Fig. 5d).

### Impact of VA nutritional status on cortical Aβ content, A Disintegrin and Metalloproteinase 10 (ADAM10) and beta-site amyloid precursor protein cleaving enzyme 1 (BACE1) enzyme activity

Through enzyme-linked immunosorbent assay (ELISA) tests, we found that low VA diet caused a significant reduction of cortical Aβ_1-40_ in APP/PS1 mice (*P* < 0.05), without affecting cortical Aβ_1-40_ content in C57 mice (Fig. 5e). There were significantly higher cortical Aβ_1-42_ content in APP/PS1 control mice than in C57 control mice (*P* < 0.05). After low VA diet treatment, cortical Aβ_1-42_ content was slightly increased in APP/PS1 mice but significantly decreased in C57 mice (*P* < 0.05). Although there was no statistical significance, cortical Aβ_1-42_/Aβ_1-40_ ratio in APP/PS1 control mice was slightly higher than C57 control mice. Low VA diet significantly and obviously increased cortical Aβ_1-42_/Aβ_1-40_ ratio in APP/PS1 mice (*P* < 0.05), but a decreased trend of this ratio was shown in C57 mice.

Results of enzyme activity test showed that APP/PS1 control mice showed lower cortical ADAM10 enzyme activity than C57 control mice (*P* < 0.05), and low VA diet caused a significant increase in cortical ADAM10 enzyme activity in APP/PS1 mice but a decrease in C57 mice (*P* < 0.05). Compared to C57 control mice, APP/PS1 control mice showed higher cortical BACE1 enzyme activity (*P* < 0.05). Low VA diet led to a further increase in cortical BACE1 enzyme activity in both APP/PS1 and C57 mice (*P* < 0.05) (Fig. 5e).

### Impact of VA nutritional status on cortical Aβ metabolism related molecule expression

The cortical Aβ metabolism related molecules expression was tested by western blotting experiment. APP/PS1 control mice showed significantly higher cortical amyloid precursor protein (APP) and C-terminal membrane-bound 99-amino acid fragment (C99) protein expression than C57 control mice (*P* < 0.05), but there was no significant difference in cortical BACE1, ADAM10, and soluble amyloid protein precursor beta (sAPPβ) protein expression between C57 control and APP/PS1 control mice. Although the APP/PS1 mice showed relative lower cortical ADAM10 protein expression, but the difference was not statistically significant. Low VA diet treatment did not affect cortical BACE1, ADAM10, and sAPPβ expression for either APP/PS1 mice or C57 mice. A reduction in cortical APP, ADAM10, and C99 protein expression was observed in low VA diet-fed APP/PS1 mice, but the difference did not reach statistical significance (Fig. 5f).

Figure 5g showed no difference in cortical t-tau and p-tau expression between C57 and APP/PS1 control mice. Low VA treatment caused a decrease in cortical t-tau protein expression in both C57 and APP/PS1 mice, although the difference was not statistically significant. The change of cortical p-tau expression and p-tau/t-tau ratio showed an opposite trend after low VA diet treatment in C57 and APP/PS1 mice, as indicated by a decreased trend in C57 mice, while an increased trend in APP/PS1 mice.

### Impact of VA nutritional status on brain glucose uptake and glucose transporters (GLUTs) expression

**Fig. 6:**
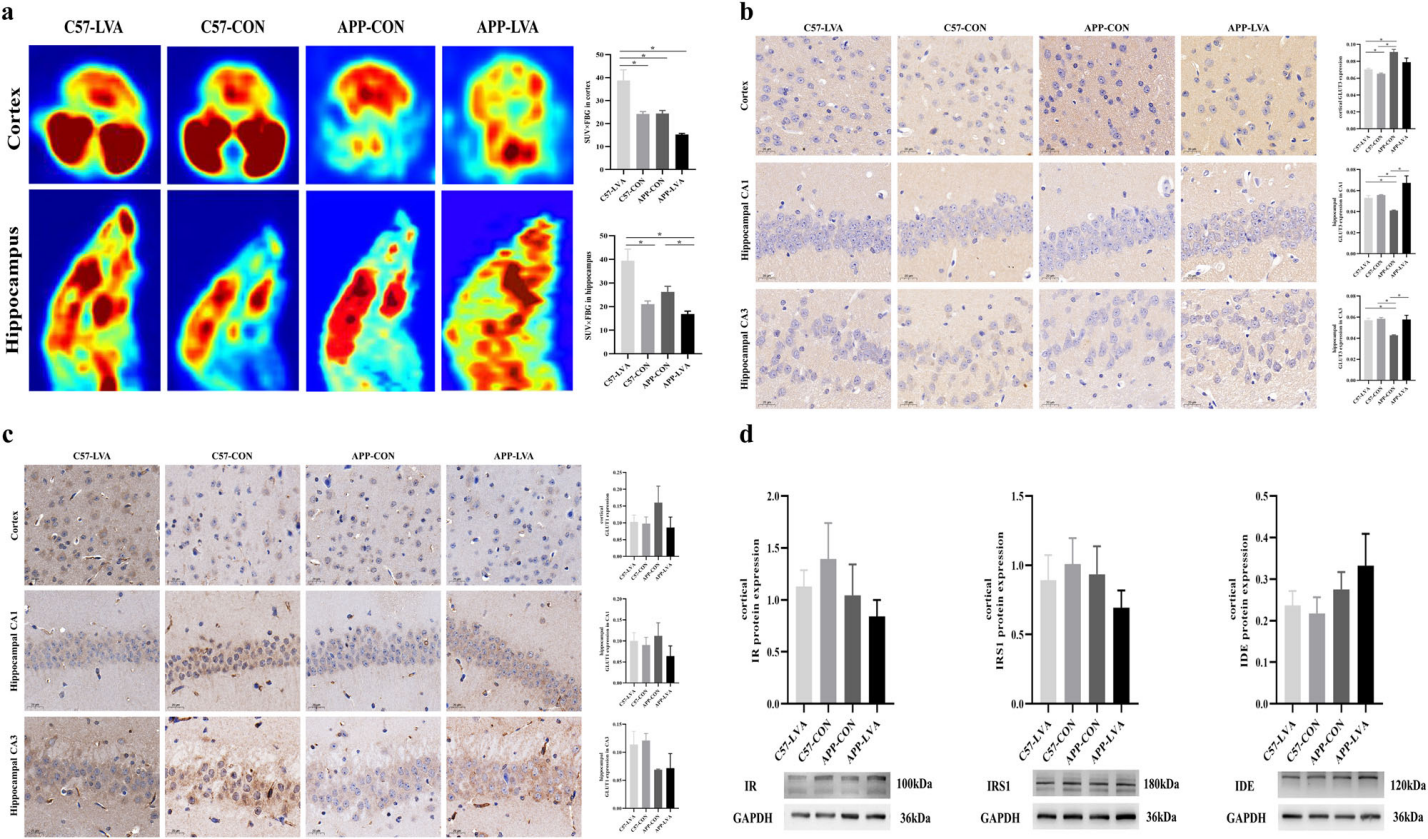
Cerebral glucose uptake capacity and glucose transport- and metabolism-related molecule protein expression in mice at the end of the experiment. **a** Results of the ^18^F-FDG-PET scan of the glucose uptake capability in both the cortex and hippocampus. **b** Brain GLUT3 expression in mice treated with different diets. **c** Brain GLUT1 expression in mice treated with different diets. **d** Molecule protein expression related with insulin metabolism in the cortex between groups. Data were expressed as mean ± SEM or mean ± SD, n = 8 in C57-CON group, n = 8 in C57-LVA group, n = 8 in APP-CON group, n = 6 in APP-LVA group. Comparisons between groups were conducted using one-way ANOVA, followed by $\check{{\rm{S}}}{\rm{id}}\acute{{\rm{a}}}{\rm{k}}$ or Games-Howell multiple comparisons test according to the homogeneity of variance. *P < 0.05. Asterisks among all histogram figures indicated significant difference of post-hoc comparison between two groups at two ends of the horizontal line.

As shown in Fig. 6a, the results of the ^18^F-FDG-PET scan indicated that APP/PS1 control mice had a glucose uptake capability similar to that of C57 control mice in both the cortex and hippocampus. The treatment of a low-VA diet caused a reduction of cortical and hippocampal glucose uptake capacity in APP/PS1 mice, and there was a statistically significant difference in the hippocampus (*P* < 0.05). In the C57 mice, glucose uptake capacity in both cortex and hippocampus was significantly enhanced after low VA diet intervention (*P* < 0.05).

Compared with C57 control mice, APP/PS1 control mice showed higher cortical glucose transporter type 3 (GLUT3) protein expression (*P* < 0.05) in the IHC assay, but lower expression in the hippocampus (*P* < 0.05). Low VA diet had no effect on hippocampal GLUT3 protein expression, but slightly up-regulated cortical GLUT3 protein expression in C57 mice (*P* < 0.05). In APP/PS1 mice, low VA diet significantly up-regulated the hippocampal GLUT3 protein expression (*P* < 0.05), but caused a slight reduction of cortical GLUT3 expression (Fig. 6b).

Results from the IHC assay showed that compared with C57 control mice, APP/PS1 control mice showed higher glucose transporter type 1 (GLUT1) expression in the cortex and hippocampal CA1 region, but lower expression in hippocampal CA3 region, although the difference was not statistically significant. The treatment of a low VA diet did not affect the cortical and hippocampal GLUT1 protein expression in C57 mice, but down-regulated the expression in the cortex and hippocampal CA1 region in APP/PS1 mice (Fig. 6c)

### Impact of VA nutritional status on insulin metabolism related molecule protein expression in the brain

As shown by the results of western blotting experiments in Fig. 6d, APP/PS1 control mice showed relatively lower cortical insulin receptor (IR) and insulin receptor substrate 1 (IRS1), but higher insulin-degrading enzyme (IDE) protein expression than C57 control mice. However, the difference between groups was not statistically significant. Low VA diet caused a slightly decrease in cortical IR and IRS1 expression in both C57 and APP/PS1 mice, but slightly up-regulated cortical IDE expression in APP/PS1 mice.

## Discussion

Previous study found that lower circulating VA level was related with the onset of dementia^23^. Additionally, the subjects with low serum VA level had a higher prevalence of MCI than the subjects with normal serum VA level^24^. Other studies also reported that the proportion of elderly adults with VA deficiency in both MCI and AD groups was higher than that in the control group^25,26^. Consistently, our data showed that the MCI subjects have lower plasma VA and lipid-adjusted VA levels than the controls, and the proportion of MCI showed a decreased trend with the increase of plasma VA or lipid-adjusted VA level in the middle-aged and elderly. We also found that following the increase of circulating VA and lipid-adjusted [VA/(TG + TC)] levels, the cognitive scores in visual & executive, language, abstract, and delayed recall domains, and the global cognition (total MoCA score) showed an increased trend. Studies have found that administration of VA could reverse the impaired hippocampal neurogenesis, reduced spatial memory, and increased anxiety-like behavior of rats with VA deficiency^27^. A population-based study also reported that plasma retinol level was positively correlated with abilities of visual & executive and memory & delayed recall^28^. These results indicated that lower circulating VA might be a risk factor for cognitive decline in the middle age and elderly.

Population-based study also highlighted VA deficiency as a potential risk factor for MCI in old individuals^24^. Another cross-sectional study conducted in the aged population also found that low serum VA level (≤ 0.200 μg/ml) caused cognition decline and increased the risk of MCI^29^. In line with these results, our data indicated that increased circulating level of retinol decreased the risk of MCI in the middle-aged and elderly population. Even after adjusting VA with lipid levels, the protective effect of high circulating VA level was consistently demonstrated. These data indicated that, for the middle-aged and elderly, a relatively higher blood VA level might predispose them to a lower risk for cognitive decline. World Health Organization (WHO) recommended that the optimal plasma VA concentration for adults was ≥ 0.30 μg/ml^30^, but there is still no recommended plasma VA level for the middle-aged and elderly to prevent cognition decline. Our data from the logistic regression and RCS analyses showed that plasma VA level at the concentration of ≥ 0.539 μg/ml was negatively associated with the risk of MCI, further indicating that a much higher plasma VA level might be beneficial to maintain normal cognitive function in the middle-aged and elderly people.

To uncover the mechanism of how VA nutritional status affects cognitive function, we also conducted a dietary intervention in AD model mice. Our data indicated that AD-like phenotype could modify the hepatic VA metabolism, as shown by increased hepatic VA level and significantly decreased hepatic RBP4 level in APP/PS1 control mice than C57 control mice. Moreover, due to homeostasis, blood retinol concentrations are tightly controlled^31^, and are not reflective of total liver VA reserves until liver reserves of VA are dangerously low^32^. Consistently, our data observed unchanged plasma VA and RBP4 levels in low VA diet-fed animals but dramatically decreased hepatic VA and increased RBP4 levels. It is also interesting to find that low VA diet caused much significant increase in hepatic RBP4 level in APP/PS1 mice as comparing with C57 mice, indicating that the AD-like phenotype might promote the remodeling of VA metabolism in the liver.

Trasino’s study observed that a 10-week VA deprivation diet significantly decreased VA concentration in the pancreas, accompanied by reduced plasma insulin and increased blood glucose levels. The histological results further confirmed the reconstruction of pancreatic endocrine cells and a decrease in the number of beta cells and insulin secretion in the islets^33^. Raja’s study also found that a 16-week low VA diet intervention caused dilated blood vessels in islets and hyperemia in islet cells^34^. Consistently, in our study, the histological changes in the pancreas were also detected in low VA diet-fed C57 and APP/PS1 mice, suggesting the critical role of VA in maintaining normal pancreatic functions. Because VA mainly stores in the liver and the damaged liver function is closely associated with metabolic dysfunction^32,35^, severe pathological changes in the liver and pancreas were observed in low VA diet-fed APP/PS1 mice compared with C57 mice, indicating that the AD model mice were more sensitive to VA deficiency-mediated liver and pancreas damage. Unfortunately, due to the small sample size, the histological changes in the liver and pancreas were not quantitatively analyzed in the present study, therefore, in the future study, quantitative comparison should be warranted to confirm the accurate histological changes.

Astrocytes, played a key role in neuroinflammation associated with AD, as well as in the production and clearance of amyloid proteins^36^. Immunoreactivity for GFAP is commonly used for labeling the intermediate filaments expressed in the cytoskeleton of astrocytes, and astrocytes with high levels of GFAP gene expression have been defined as ‘disease-associated astrocytes’ in AD rodent model. There was also increased GFAP expression in the brain of AD patients^37^. Similarly, increased GFAP expression in the cortex of AD model mice was found higher than that of wildtype mice, with morphological changes in this study. Moreover, low VA diet induced higher expression of GFAP in both wildtype and AD model mice, especially in APP/PS1 mice, indicating that there was exacerbated neuroinflammation in AD pathology, and low VA diet might aggregate this inflammatory pathology. In the present study, low VA diet induced the deficit in nesting behavior in AD model mice. The nesting behavior was multi-brain-dependent spontaneous ability and was considered similar to activities of daily living (ADL) skills and executive function^38,39^. Additionally, the comprehensive histopathological damage caused by VA deficiency was observed, including increased cerebral Aβ plaques, activated astrocytes, distinct atrophied neurons and nuclear deformation. Senile plaque is the typical pathology of AD, over-accumulation of senile plaque in the brain will produce neurotoxic effects, ultimately resulting in cognitive decline and the onset of dementia^24^. All these data suggested that low VA diet might promote the formation of senile plaques, accelerate the neuroinflammatory damage and the progression of AD pathology, and ultimately caused deficit in behavior.

In addition, APP-CON mice have much higher cortical Aβ_1-42_ content than C57-CON mice, suggesting that abnormal Aβ metabolism might be attributed to the discrepant ratio of Aβ_1-42_/Aβ_1-40_, as the sensitive and specific index for AD neurological pathology^40^. After low VA diet treatment, the cortical Aβ_1-42_/Aβ_1-40_ ratio in APP/PS1 mice was further increased, in contrast to a slight decrease in C57 mice. Abnormal APP cleavage plays an essential role in the pathology of AD^41^, which the BACE1-cleaved C99 can be further cleaved by γ-secretase to yield different Aβ species^42^. An increased BACE1 expression was observed in the brain of AD model mice with marginal deficiency or deficiency of VA^8,28^. Additionally, most studies have shown that VA supplement could reduce Aβ generation by increasing the expression and activity of ADAM10 in the brain^27,43,44^. These data suggested the potential regulatory effect of VA deficiency on cerebral Aβ generation. We found that low VA diet treatment caused a dramatic increase in cortical BACE1 enzyme activity in both APP/PS1 mice and C57 mice. Moreover, a discrepant regulatory effect of low VA diet on cortical ADAM10 enzyme activity was observed between C57 and APP/PS1 mice. We speculated that the increase in ADAM10 enzyme activity in low VA diet-fed APP/PS1 mice might be an abnormal self-compensatory mechanism to rescue abnormal Aβ cleavage in this AD model mice. Although a low VA diet did not affect the protein expression of cortical APP, C99, and sAPPβ in both APP/PS1 and C57 mice, our data implied that the net regulatory effect of a low VA diet on ADAM10 and BACE1 enzyme activities might determine the balance between non-amyloidogenic and amyloidogenic cleavage of APP, as well as the production of Aβ.

T-tau and p-tau are critical elements of AD pathophysiology, and the increased ratio of p-tau to t-tau in cerebrospinal fluid is another well-known biomarker for AD^29^. In our study, the discrepant reaction of cerebral tau metabolism to low VA diet in C57 and APP/PS1 mice, as indicated by the change of cortical p-tau expression and p-tau/t-tau ratio, suggested that a low VA diet aggravated the dysregulation of the self-protective compensatory mechanism and AD-like phenotype in APP/PS1 mice, further promoting the development of AD^8^.

Insufficient cerebral glucose concentration leads to impaired growth and development of neurons, contributing to the onset of AD^45^. Zhang et al. reported that a VA free diet caused the activation of the glycolytic pathway with elevated brain glucose metabolism in the cortex of C57 mice, suggesting a self-defense glucose mechanism of neurons to low VA nutritional status^46^. Consistently, we found that low VA diet-fed C57 mice displayed enhanced brain glucose uptake capacity than control diet-fed mice. In contrast, APP-LVA mice showed decreased hippocampal glucose uptake capacity, accompanying with dramatic increase in hippocampal GLUT3 expression. GLUT3 is the most abundant glucose transporter, and mainly exists on axons and dendrites^47^. The expression and distribution of GLUT3 protein are closely related to local brain glucose demand^47^. A previous study has reported the regulatory effect of VA on glucose transporter expression^48^, for example 9-cis-retinoic acid could reduce GLUT2 to attenuate the glucose-stimulated insulin secretion (GSIS) in islets^49^. In our study, although hippocampal GLUT3 protein was increased in low VA diet-fed APP/PS1 mice, the brain glucose uptake capacity was dramatically decreased. These results indicated a destruction of self-defense capacity in APP/PS1 mice to rescue abnormal glucose metabolism homeostasis under low VA nutritional status. The unchanged expression of GLUT1 in low VA diet-treated mice further confirmed that GLUT3 was a primary VA-targeted molecular in affecting glucose uptake and metabolism in the brain. Moreover, the different responses of cerebral glucose uptake capacity and expression of GLUTs between C57 and APP/PS1 mice also implied a weakened self-regulatory ability of AD model mice to antagonize the low VA diet-mediated abnormal brain glucose metabolism.

Insulin resistance might be associated with inflammation and metabolic disorders in the brain and involved in the development of AD^50^. The role of IR, IRS, and IDE in affecting the occurrence and development of AD through regulating central energy metabolism and Aβ clearance has been reported by previous studies^51,52^. A significant reduction in the level of IRS1/2 was observed in the temporal cortical neurons in AD mice^53^. In line with these results, our study found decreased trend of IR and IRS1 expression and increased trend of IDE expression in the cortex of APP/PS1 mice compared with C57 mice, indicating that APP/PS1 mice are prone to have cerebral insulin metabolism disorder^54^. As an insulin-degrading proteolytic enzyme, IDE also plays a vital role in the degradation of Aβ in the brain. It has been reported that cerebral IDE level and its activity decreased simultaneously during insulin resistance, while increased insulin level induced competitive binding of IDE with Aβ, resulting in reduced Aβ clearance and excessive accumulation in the brain^55^. After low VA diet treatment, the expression of both IR and IRS1 showed a decreased trend in both C57 and APP/PS1 mice, but the expression of IDE displayed an obviously increased trend in APP/PS1 mice. Studies have shown that VA could increase insulin sensitivity by enhancing insulin signaling or triggering insulin release, and VA deficiency caused an elevation of cellular retinol-binding protein in islets and disturbed insulin release^56^. In agreement with these results, our data indicated that the low VA diet might aggravate cerebral insulin resistance and inhibit glucose uptake in AD model mice. These data showed the potential impact of a low VA diet on the brain insulin metabolism signaling pathway. Moreover, considering the potential role of IDE in cerebral Aβ clearance, we speculated that IDE might play an intermediary and linkage role in glucose and Aβ metabolism in VA-deficiency accelerated progression of AD.

In conclusion, higher plasma VA level was beneficial to maintaining better cognitive performance in the middle-aged and elderly population, and plasma VA at the concentration of ≥ 0.539 μg/ml might decrease the risk of MCI in middle-aged and elderly individuals. Low VA nutritional status might impair the liver and pancreas to disrupt the insulin signaling pathway and glucose uptake and transport in the brain, and increase Aβ_1-42_/Aβ_1-40_ and p-tau/t-tau ratios, further promoting the senile plaque deposit and aggregating cerebral neuroinflammation, finally to exacerbate AD pathology and cause nesting behavior disorder.

## Methods

### Participants

Two thousand two hundred and two adults aged 50 years and older were recruited from both Wulituo and Nanyuan communities and the medical examination center of Guang’anmen hospital, in Beijing, China. According to previous studies^57–59^, the estimated average plasma VA level was 0.57 ± 0.20 and 0.62 ± 0.19 μg/ml in the MCI and non-MCI middle-aged-and-older adults, respectively. The two-side *α* and statistical power (1 - *β*) was set at 0.05 and 0.90, respectively, and the sample size ratio between MCI and control groups was set at 1:2. Meanwhile, hypothesizing the loss of follow-up and elimination rate of 10%, the study was expected to include 273 and 546 participants in MCI and control groups at least, respectively. In addition, in light of that previous investigation have suggested the prevalence of MCI among the Chinese adults aged 55 years and older was 14.5%^60^, therefore, there were 1883 participants aged 50 years and older required to be recruited in the study. The inclusion criteria for recruitment were as follows: ① participants aged 50 years and older; ② those who signed informed consent; ③ those without central nervous system diseases, such as Parkinson’s disease, active epilepsy, lewy body dementia, subdural hematoma, or meningitis; ④ those without severe sensory and perceptual disorders that prevented them from completing cognitive function and dietary investigations; ⑤ participants who were able to complete the questionnaire or undergo measurements for anthropometric and dietary investigation.

Participants were excluded as the following: ① not completing the questionnaire survey or not retaining medical examination results or blood sample (n = 13); ② without diabetes but FBG level at ≥ 6.1 mmol/L (n = 292); ③ missing reporting results in questionnaire investigation, medical examination or laboratory tests (n = 80). Finally, 1817 subjects aged 50 years and older were included in the study, of which the sample size in MCI and control groups was 679 and 1138, respectively, with meeting the criteria of the least sample size (MCI group: n = 273; control group: n = 546). The study was conducted following the Declaration of Helsinki, and the study protocol has been approved by the Medical Ethics Review Committee of Capital Medical University (No.2012SY23). Informed consent was obtained from all subjects before participating in the study.

### Demographic and dietary survey

The self-designed questionnaire described in the previous study^61^ was used to investigate the subjects’ social demography and disease history. The questionnaire included age, gender, body mass index (BMI), education level (illiterate, primary school, junior high school, high school, junior college, undergraduate and above), physical activity (never, 1–3 days/week, 4–6 days/week, every day), living alone (yes or no), reading habits (yes or no), using TV and computer (yes or no), smoking (never, ever, current), drinking alcohol (yes or no), usage of dietary supplements (yes or no), AD family history (yes or no), disease history [including type 2 diabetes mellitus (T2DM), hyperlipidemia, stroke, chronic kidney disease]. The measured indexes included height and weight. BMI was calculated as weight (kg)/height (m)^2^.

Food Frequency Questionnaire (FFQ) was used to collect information on daily dietary intake. The food items included cereal, vegetable, fruit, dairy, legume, red meat, poultry meat, fish, egg, whole grain, and cooking oil. The intake of cooking oil was calculated on average by the number of family meals and members and the monthly consumption of cooking oil. The adjusted dietary balance index (DBI) was calculated according to the Revision of Dietary Balance Index of China: DBI_16. The modified DBI_16 consists of 12 food items: cereal, vegetable, fruit, dairy, legume, red meat, poultry meat, fish, egg, whole grain, cooking oil, and food diversity. DBI_16 scores were calculated for each food item. LBS, HBS, and diet quality distance (DQD) were also calculated. The absolute value of the sum of all negative scores indicating an insufficient extent of dietary intake is defined as LBS. HBS is sum of all positive scores indicating an excessive amount of dietary intake. DQD is the sum of HBS and LBS, indicating the overall extent of dietary imbalance^62^.

### Cognitive function measurement

The MoCA scale was used to evaluate the subjects’ cognitive performance. The test was conducted by uniformly trained nurses and doctors from the community health service center. According to the reported conclusion of the cognitive function screening in Chinese elderly^61^, the cut-off values of MoCA score for MCI were as follows: ≤ 13 for illiterate, ≤ 19 for those with no more than 6 years of education, and ≤ 24 for those with more than 6 years of education, respectively.

### Blood sample collection and parameter measurement

Fasting peripheral venous blood (8 ml) was taken in the morning. Plasma samples were separated and stored in a -80°C refrigerator for parameter measurement. FBG, TG, and TC levels were measured by the automatic biochemical analyzer. Plasma HDL-c level was measured by a commercially available analytical kit from the Instrumentation Laboratory (Lexington, WI, USA). Low-density lipoprotein cholesterol (LDL-c) was calculated according to the Friedewald formula^63^. The plasma VA (retinol) level was determined by high-performance liquid chromatography (HPLC) according to the method described by a previous study^64^. All samples for each participant were analyzed within a single batch, and the inter-assay coefficients of variation (CV) were less than 5%. The plasma VA nutritional status was defined as deficiency (< 0.2 μg/ml), marginal deficiency (0.2 – 0.3 μg/ml), and sufficiency (≥ 0.3 μg/ml), respectively, according to the WHO guideline^30^.

### Animals and treatments

Sixteen male 3-week-old C57BL/6J wild-type mice, and fourteen male 3-week-old APP/PS1 mice were purchased from Beijing Vital River Laboratory Animal Technology Co., Ltd. (Beijing, China). The mice were given a standard diet for one week to adapt to the environment, and then randomly divided into the control diet (CON) group fed with a standard diet containing normal VA (4000 IU/kg), and low VA diet (LVA) group fed with a standard diet with low VA (400 IU/kg), according to baseline blood glucose and body weight: ①C57-LVA group (n = 8); ②C57-CON group (n = 8); ③APP-CON group (n = 8); ④APP-LVA group (n = 6). The diet composition of each group is shown in Table 3. The intervention feed was customized by Changzhou SYSE Co., LTD (Changzhou, China). All animals were housed at room temperature of 20–23 °C under a 12-h light-dark cycle and provided with free water and feed access. The dietary intervention was conducted for eight months to nine months of age corresponding to the middle adulthood of human^65^, in accordance with guidelines for the Care and Use of Laboratory Animals in China, as approved by the Medical Ethics Review Committee of Capital Medical University (No. AEEI-2020-012).

**Table 3:** Composition of experimental diets (g/kg)

Nutrients in diet	Groups	
	CON-diet	LVA-diet
**Ingredient (g/kg)**		
*Casein*	200	2
*L-Cystine*	3	3
*DL-Methionine*	3	3
*Corn Starch*	650	650
*Fructose*	0	0
*Maltodextrin*	37.5	37.5
*Sucrose*	0	0
*Cellulose*	50	50
*Palm oil*	0	0
*Corn oil*	50	50
*Soybean oil*	0	5
*Lard*	0	0
*Mineral Mix*	35	35
*Choline bitartrate*	2	2
*Vitamin D (IU)*	1000	1000
*Vitamin A acetate (IU)*	4000	400
*Vitamin E acetate*	13.2	13.2
**kcal%**		
*Protein*	20.8	20.8
*Carbohydrate*	67.7	67.7
*Fat*	11.5	11.5

### Oral glucose tolerance test

After the dietary intervention, OGTT test was conducted according to the previously described method^66^. Briefly, after a 12-h fasting period, the baseline blood glucose levels of all animals were measured, followed by administering 1 g/kg of glucose through oral gavage. Then a standard glucometer (Accu-Chek, Roche) was used to measure blood glucose levels at 30, 60, and 120 minutes post-glucose administration, with blood samples collected from the tail tip.

### Behavioral testing

After dietary intervention, nesting behavior was tested to measure the nesting capacity of animals, and the Morris water maze (MWM) test was applied to measure the spatial learning and memory of animals. In the MWM test, the escape latency from entering the water to finding the round platform within 90 s and the number of times across the platform from entering the water within 90 s were recorded with a video tracking system (Water Maze 2.6 Institute of Materia, Chinese Academy of Medical Sciences DMS-2, Beijing, China). In the light-dark box test, using the darkening characteristic of mice, the memory level of mice was tested by recording the number of times to enter the camera obscura (i.e. number of errors). In the NOR test, the NOR index was calculated to measure animals’ long- or short-term memory. Results were calculated individually for each animal.

### Brain glucose uptake

Positron emission tomography (PET) was used to measure mouse brain glucose uptake according to the previous method^67^.

### Tissue preparation

After dietary intervention, the mice were anesthetized with 1.5% pentobarbital of 0.1 ml per 20 g body weight through intraperitoneal injection to the unconscious state, then followed by euthanasia with carotid artery bleeding. Blood was collected through the carotid artery into a BD anticoagulant tube containing heparin lithium (BD, America). The plasma was separated and collected in a 1.5 ml EP tube and stored at –80 °C for biochemical testing. The brain tissues were dissected immediately and separated along the middle sagittal sulcus. The left hemisphere was fixed for immunohistochemistry examination. The right hemisphere’s cortex and hippocampus were immediately separated and stored in a -80°C for western blotting and biochemical analysis.

### Measurement of retinol, RBP4, insulin, Aβ_1-40_ and Aβ_1-42_ levels, and enzyme activity of cerebral BACE1 and ADAM10 in mice

HPLC method was used to measure plasma and liver retinol content according to the previously published method^68^. ELISA assay kits (Immunoway, China) were used to measure plasma insulin level, both plasma and hepatic RBP4 levels according to the manufacturer’s instructions, and ELISA assay kits (Novus Biologicals) for cortical Aβ_1-40_ and Aβ_1-42_ levels. Assay kits from BioVision (UK) and AnaSpec (USA) were applied to test the enzyme activity of cerebral BACE1 and ADAM10, respectively.

### Measurement of brain morphological changes of neurons and Aβ plaque

Glycine silver staining was applied to observe the morphological changes of neurons.

Aβ plaques in the brain were measured by using a Congo red (Key Gen Bio Tech, Nanjing, China) staining method according to the description of Oksman^30^.

### Histochemical and immunohistochemical staining

The liver and pancreas samples were embedded in a 4% polyformaldehyde fix solution, and subsequently embedded in paraffin blocks. The paraffin blocks were then sectioned into slices of 5 μm and stained with hematoxylin-eosin (HE) according to previously described methods^69^. IHC assays were performed on brain paraffin-embedded sections using antibodies directed against GLUT1 (1:100 dilution, cell signaling technology, USA), GLUT3 (1:200 dilution, cell signaling technology, USA), and GFAP (1:1200, Servicebio, China). A horseradish peroxidase (HRP)-labeled secondary antibody (1:200) was used for positive detection and diaminobenzidine as a chromogen. Then, the section was observed and photographed by an electric microscope (Olympus BX61, Japan).

### Western blotting

About 30 mg of the tissue was homogenized in 0.6 ml of protein lysis buffer [radioimmunoprecipitation assay (RIPA): phenylmethanesulfonyl fluoride (PMSF) = 100:1; the final concentration of PMSF was 1 mM]. The homogenized tissue was transferred into a 1.5 ml EP tube and centrifuged at 13500 rpm/min at 4 °C for 10 min. The supernatant was used for protein concentration measurement. Protein concentrations were determined by bicinchoninic acid (BCA) protein assay. A total of 30 μg of protein were resolved on polyacrylamide gels (Bio-Rad, USA). After transferring to polyvinylidene fluoride (PVDF) membranes, the membranes were blocked in tris-buffered saline (TBS), 0.1% Tween 20, and 5% nonfat powdered milk for 1 h, followed by an overnight incubation with primary antibody. The used primary antibodies were as follows: rabbit monoclonal anti-β-actin (1:1000, Servicebio, China), anti-glyceraldehyde-3-phosphate dehydrogenase (anti-GAPDH) (1:1000, ZSGB-BIO, China; 1:1000, Servicebio, China), anti-BACE1 (1:1000, Abcam, UK), anti-APP (1:1000, Abcam, UK), anti-sAPPβ (1:1000, BioLegend, USA), anti-C99 (1:2000, EMD Millipore, Germany), anti-ADAM10 (1:500, Servicebio, China), anti-tau (1:1000, proteintech, China), anti-p-tau (1:1000, Abcam, UK), anti-IDE (1:5000, Abcam, UK), anti-IR (1:1000, Abcam, UK), and anti-IRS1 (1:1000, Servicebio, China). Then, the membrane was washed in TBST and incubated with HRP-goat-anti-rabbit secondary antibody (1:5000, Immunoway, China) or HRP-goat-anti-mouse (1:5000, Immunoway, China) for 1 h. Protein bands were visualized by chemiluminescence (ECL western blotting substrate, Thermo Scientific, USA) using a FUSION-FX imaging system (Vilber Lourmat, France). FusionCapt software (Vilber Lourmat, France) was applied for quantitative analysis of protein bands.

### Statistical analysis

In the population study, IBM SPSS v.23.0 and R v.4.0.3 were used for statistical analysis. Continuous data were expressed as mean ± standard deviation (SD) or median (quartile 25th, quartile 75th), and student’s *t* test and Mann–Whitney U test were used for comparison between groups. Categorical data were expressed as number and percentage, and chi-square test and Fisher’s exact test were used for comparison between groups. According to the quintiles of plasma VA level, the participants were divided into five groups. The cut-off points of plasma VA and lipid-adjusted VA levels were listed in Supplemental Table S1. GLM was applied to compare plasma VA and lipid-adjusted VA levels between MCI and control groups, with adjustment of age, gender, BMI, physical activity, smoking, drinking alcohol, usage of dietary supplement, hyperlipidemia (not adjusted if compared plasma VA/(TC + TG) levels), stroke, chronic kidney disease, T2DM, LBS, and HBS. The GLM was also used to compare cognitive functions across quintiles of plasma VA and lipid-adjusted VA levels with adjustment of age, gender, BMI, education, physical activity, living alone, reading habits, using TV and computer, smoking, drinking alcohol, usage of dietary supplements, AD family history, hyperlipidemia (not adjusted for lipid-adjusted VA levels), stroke, chronic kidney disease, T2DM, LBS, and HBS. Logistic regression was conducted to investigate the association between plasma VA, lipid-adjusted VA and the risk of MCI, respectively, with the lowest quintile as a reference. The trend test across quintiles was conducted by entering the categorical plasma VA variable as a continuous variable into models. In model 1, we adjusted for age, gender, BMI, education, physical activity, living alone, reading habits, using TV and computer, smoking, drinking alcohol, usage of dietary supplements, AD family history, T2DM, hyperlipidemia (not adjusted for lipid-adjusted VA levels), stroke, chronic kidney disease. Model 2 included adjustment for confounding factors in model 1 with additional adjustment for LBS and HBS. Model 3 was further adjusted by DQD based on model 1. Since the association in the three models described above were nearly identical, the dose-response relationship between plasma VA level and risk of MCI was analyzed using RCS curve with four knots at the 20th, 40th, 60th, and 80th percentiles after adjustment for covariates in model 2. Two-side *P* value < 0.05 was considered statistically significant.

In animal experiments, data were expressed as mean ± standard error of the mean (SEM) or mean ± SD. GraphPad Prism 8.0 (GraphPad Software Inc., San Diego, CA, USA) was used for graphic display of data. The data analysis was performed by IBM SPSS v23.0. Comparisons between groups were conducted using one-way analysis of variance (ANOVA), followed by $\check{{\rm{S}}}{\rm{id}}\acute{{\rm{a}}}{\rm{k}}$ or Games-Howell multiple comparisons according to the homogeneity of variance. Two-side *P* < 0.05 was considered statistically significant.

## Supplementary information

Supplemental files

## Data Availability

The datasets used and/or analyzed during the current study available from the corresponding author on reasonable request.
