# Supplementary material for: Impact of vitamin A on aged people’s cognition and Alzheimer’s disease progression in an animal model
Source: NPJ Sci Food. 2025 May 8;9:67. doi: 10.1038/s41538-025-00402-1 (PMC12062344; doi:10.1038/s41538-025-00402-1)
Supplement: Supplementary file 1 — Supplemental files [file 41538_2025_402_MOESM1_ESM.pdf]

Supporting information

Supplemental Table S1. The quintiles of plasma VA and lipid-adjusted VA levels

Parameters	Q1	Q2	Q3	Q4	Q5
VA	≤ 0.386	0.387 – 0.463	0.464 – 0.538	0.539 – 0.655	≥ 0.656
(μg/ml)	0.332 ± 0.048	0.425 ± 0.023	0.501 ± 0.023	0.596 ± 0.033	0.789 ± 0.113
VA/(TC+TG)	≤ 0.057	0.058 – 0.068	0.069 – 0.085	0.086 – 0.106	≥ 0.107
(μg/μmol)	0.048 ± 0.008	0.063 ± 0.003	0.076 ± 0.005	0.095 ± 0.006	0.132 ± 0.022

Data were expressed as mean± sd.

Statement

The results of western blotting listed in this paper are the results of animal diet intervention experiments carried out by the research group in the early stage by giving C57BL/6J wildtype and APP/PS1 mice FAT or control diet and different dose levels of vitamin A (VA). C57BL/6J wildtype and APP/PS1 mice were randomly divided into eight intervention groups according to baseline blood glucose and bodyweight: ① C57-FAT: C57BL/6J mice feed by high-fat diet (HFD) and normal VA group (a standard diet with high-fat level but normal level of VA); ② C57-FAT-LVA: C57BL/6J mice feed by HFD and low VA (a standard diet with high-fat level but a lower level of VA); ③ C57-CON: C57BL/6J mice feed by normal control diet (a standard diet with normal level of VA); ④ C57-LVA: C57BL/6J mice feed by low VA diet (a standard diet with a lower level of VA); ⑤ APP-FAT: APP/PS1 mice feed by HFD diet and normal VA group (a standard diet with high-fat level but normal level of VA); ⑥ APP-FAT-LVA: APP/PS1 mice feed by HFD and low VA (a standard diet with high-fat level but a lower level of VA); ⑦ APP-CON: APP/PS1 mice feed by normal control diet (a standard diet with normal level of VA); ⑧ APP-LVA: APP/PS1 mice feed by low VA diet (a standard diet with a lower level of VA). The component of the standard diet was based on AIN-76A with a normal VA concentration of 4000 IU/kg. The low VA diet containing VA at the dosage of 400 IU/kg and the HFD contained 45% fat (W/W) and 20% fructose (W/W) were used for dietary intervention.

In order to make the results look concise and intuitive, the protein band results presented this time are the four groups of protein results related to this article (i.e. C57-CON, C57-LVA, APP-CON, APP-LVA) selected from the eight groups of results, but do not present all the band results. Therefore, segmentation lines were used to distinguish some protein bands. The original pictures of the presented results are as follows. The original data and experimental records of the experimental results can be checked and reviewed at any time.

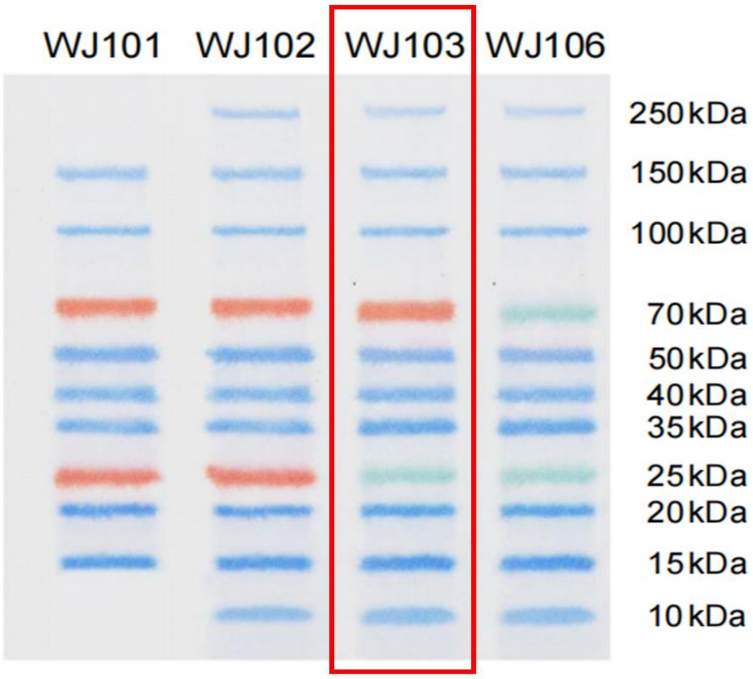

Supplemental Fig 1 Schematic diagram of the protein marker

This is a schematic diagram of the protein marker we use (Cat. No. WJ103). https://www.epizyme.cn/pro_show.aspx?productid=1073

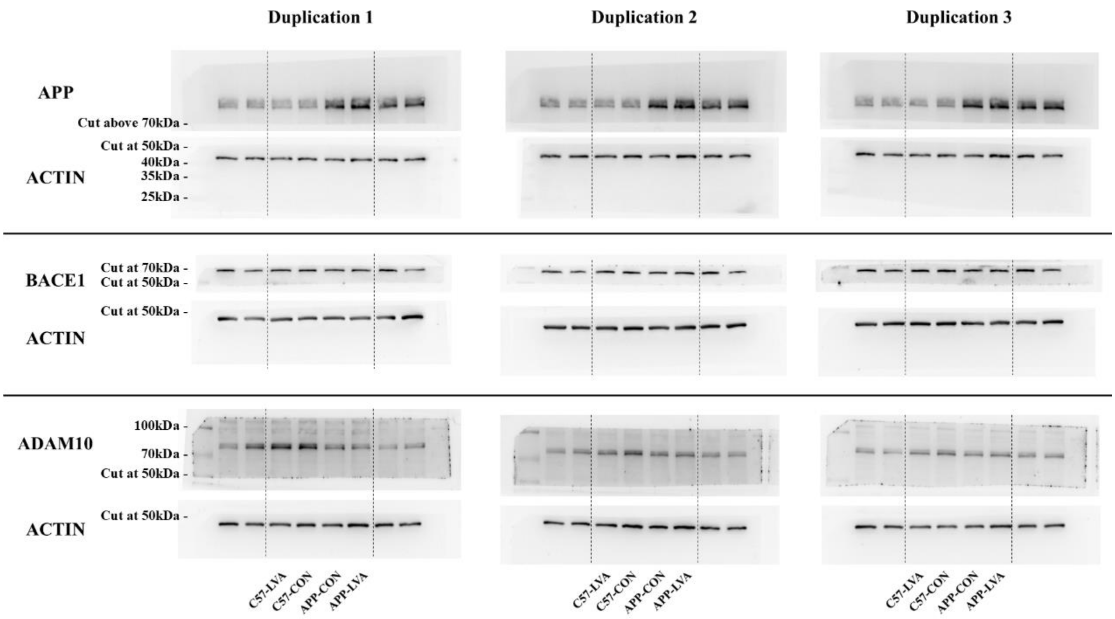

Supplemental Fig 2 Western blot showing cortical APP, BACE1, and ADAM10 expression with three repeated measurements in four groups

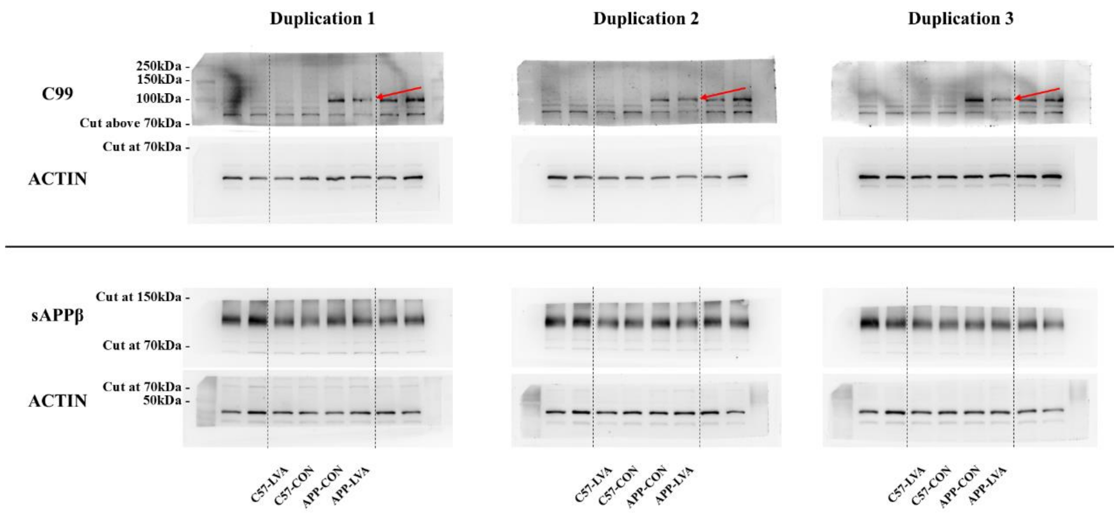

Supplemental Fig 3 Western blot showing cortical C99 and sAPPβ expression with three repeated measurements in four groups

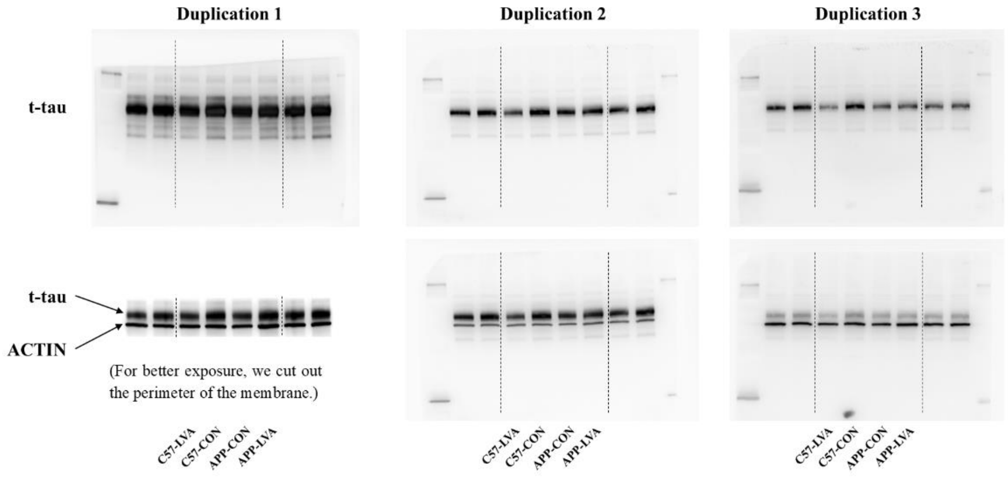

Supplemental Fig 4 Western blot showing cortical t-tau expression with three repeated measurements in four groups

Note: Since the molecular weight of t-tau is very close to that of ACTIN, we first incubate t-tau on the whole membrane, and then wash the membrane and re-incubate ACTIN after exposure. As shown in the figure, the upper figures show the result of first exposing t-tau, and the lower figures correspond to the result after adding ACTIN.

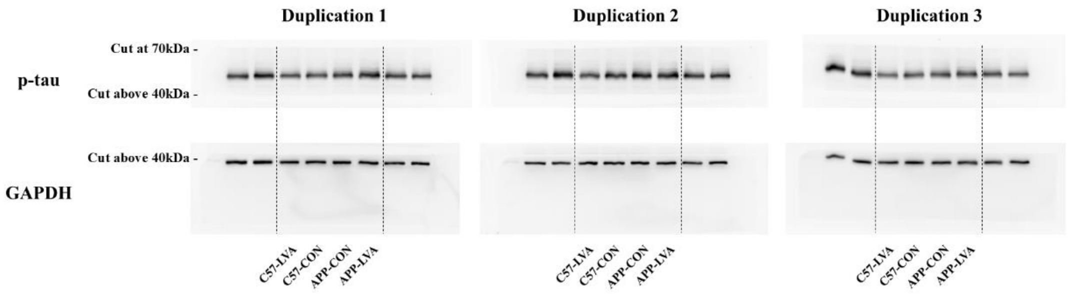

Supplemental Fig 5 Western blot showing cortical p-tau expression with three repeated measurements in four groups

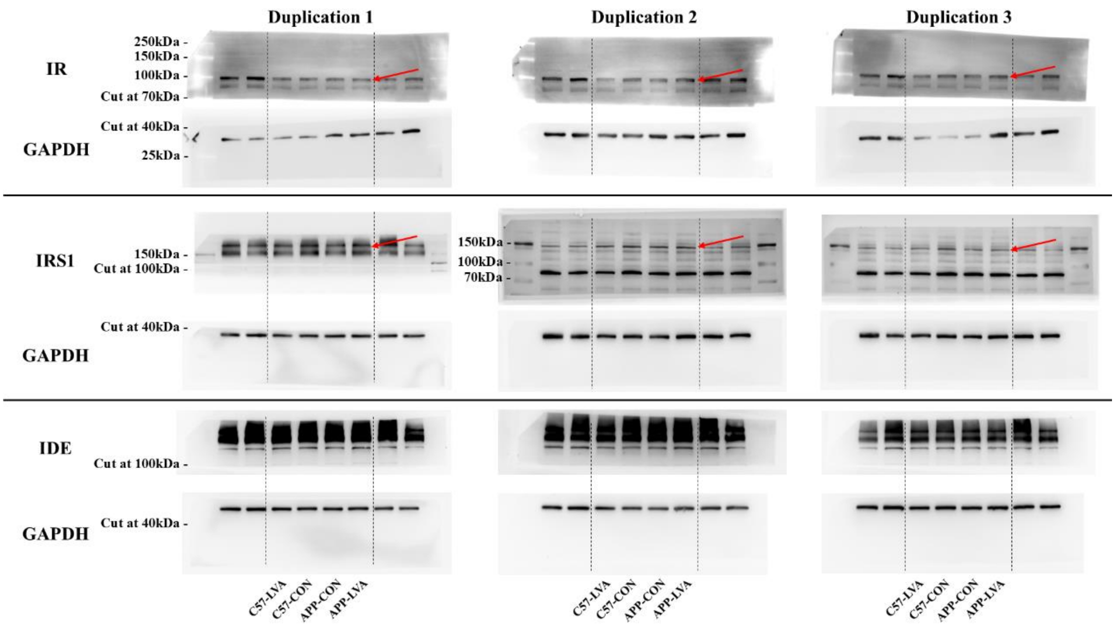

Supplemental Fig 6 Western blot showing cortical IR, IRS1, and IDE expression with three repeated measurements in four groups
